# Small Extracellular Vesicles Loaded with Immunosuppressive miRNAs Leads to an Inhibition of Dendritic Cell Maturation

**DOI:** 10.1007/s00005-022-00664-7

**Published:** 2022-11-01

**Authors:** Liliana Czernek, Łukasz Pęczek, Markus Düchler

**Affiliations:** Department of Bioorganic Chemistry, Centre of Molecular and Macromolecular Studies, Polish Academy of Sciences, Lodz, Poland

**Keywords:** Dendritic cells, Electroporation, miRNAs mimics, Tolerogenic, Extracellular vesicles

## Abstract

In particular conditions, inhibition of an immune response is required to prevent tissue damage. Among these conditions are diseases caused by an over-reactive immune response, such as autoimmune or allergic disorders, or imminent organ rejection after transplantation. To avoid tissue damage, drug-mediated systemic immune suppression is an option, but it comes with high costs in the form of susceptibility to viral and bacterial infections. Thus, the induction of antigen-specific tolerance is preferable. Extracellular vesicles (EVs) are capable of delivering antigen together with immunosuppressive signals and may be used to specifically induce antigen-specific tolerance. However, naturally occurring EVs are heterogeneous and not all of them show immunosuppressive character. In our trials to engineer cell culture derived EVs to increase their tolerogenic potential, we equipped them with immunosuppressive miRNA mimics. Small EVs (sEVs) were isolated and purified from the human monocytic THP-1 cell line or from healthy donor peripheral blood mononuclear cells, and electroporated with miR-494 and miR-146a mimics. The acquired immunosuppressive potential of the modified sEVs was demonstrated by their ability to alter the major histocompatibility complex molecules and co-stimulatory receptors present on dendritic cells (DCs). To avoid allogeneic responses, the same cells that produced the sEVs served also as recipient cells. In contrast to the treatment with unmodified sEVs, the tolerogenic sEVs impeded lipopolysaccharide-induced maturation and kept DCs in a more immature developmental stage. Our experiments show that simple manipulations of sEVs using immunosuppressive cargo can lead to the inhibition of DC maturation.

## Introduction

To avoid healthy tissue destruction, the activity of cytotoxic *T* lymphocytes (CTLs) needs to be firmly controlled. Antigen-presenting cells (APCs) are the central decision makers about destructive immune responses versus tolerance. Among APCs, the dendritic cells (DCs) are one of the key players for the initiation of adaptive immunity and the stimulation of CTLs. DCs capture pathogen protein antigens, which are degraded; the resulting peptides are presented on major histocompatibility complex class II (MHC II) molecules. Recognition of these MHC II complexes by antigen receptors of helper *T* cells is required for the establishment of adaptive immunity. The MHC complex, also known as human leukocyte antigen (HLA) system, consists of two classes of molecules, MHC I and MHC II. MHC I complexes are present on all cells and present peptides to *T* cell receptors to either expose pathogen fragments for CTL activation, or to induce immune tolerance by exposing self-peptides (Janelle et al. 2020; Waldman et al. 2020). For the induction of effective immunity, the cooperation of several types of cells and molecules is required. Activation of CTLs needs direct contact with APCs and with T helper cells. Obligatory signals are delivered through co-stimulatory receptors, such as CD40, CD80, and CD86, and through cytokines, such as interleukin (IL)-12.

CTLs are indispensable to keep an organism free from cancerous cells. CTLs recognise tumour cells when they expose tumour-associated antigens (TAA) via MHC I complexes on their surface. TAAs are either mutated cellular proteins, of molecules with altered post-translational modifications (de Aquino et al. 2015). To survive and proliferate, tumours need to escape the destruction by the attacks of the immune system. To avoid the cytotoxic T lymphocyte response, cancer cells may down-regulate MHC expression. Furthermore, tumour derived factors target the executory capacity of the immune system to impair proper immune response activation (Czernek and Düchler 2017). The anti-cancer immune response may be also inhibited by the production of immune suppressive cytokines by cancer cells or other cells in the tumour microenvironment. Transforming growth factor (TGF)-β and IL-10 are among the most potent immunosuppressive cytokines. Another way to induce immune tolerance is based on the down-regulation of co-stimulatory molecules on APCs. Lack of appropriate co-stimulation after the engagement of the *T* cell receptor induces anergy or tolerance in *T* cells (Cuenca et al. 2003). Elimination of tumour-specific cytotoxic *T* cells (CTLs) happens also by apoptosis through engagement of death receptors by their fitting ligands. Finally, cancer-induced immunosuppression enforces in the induction and activation of immune suppressor cells including regulatory *T* (Treg) cells and myeloid-derived suppressor cells (MDSCs).

The communication between cancer cells and cells of the immune system uses a variety of information carriers, among them extracellular vesicles (EVs). The three major classes of EVs are exosomes, microvesicles (ectosomes) and apoptotic bodies. Exosomes are nanovesicles of about 30–150 nm in size, that are generated constitutively by all cell types (Gurunathan et al. 2019a; Kalluri and LeBleu 2020; Petgel and Gould 2019). Exosomes contain and transport proteins, lipids, mRNA and miRNAs (Kim 2019). They are generated intracellularly in multivesicular bodies and released into the cellular environment. When taken up by other cells, functional mRNA and microRNA (miRNA) can reprogram recipient cells by influencing the protein expression levels. For instance, exosomes can promote immunity by regulating signals for both, adaptive and innate immune responses (Zhang et al. 2014). Exosomes produced by APCs can activate *T* cells, leading to increased immune responses (Robbins and Morelli 2014; Sprent 2005). However, exosomes are not simple molecules delivering plain messages. Due to their complex and variable composition and the intricate interaction with the target cells, the outcome of exosome signalling is highly variable. Cancer derived exosomes, for instance, are not only able to stimulate but also to suppress immune responses, especially in late stages of progression.

Cancer cell-derived exosomes can contribute to cancer immunosuppression through reprogramming of APCs converting them into repressor cells (Chen et al. 2018; Czernek and Düchler 2017; Düchler et al. 2019; Robbins and Morelli 2014). Their cargo includes efficient inhibitors of immune stimulation, such as immunosuppressive cytokines and miRNAs which enforce the induction of tolerance (Stumpfova et al. 2014; Sun et al. 2019; Tang et al. 2018, 2020). When the maturation of DCs is prevented, tolerogenic immature or semi-mature DCs arise which can convert naïve *T* cells into Treg cells. Treg cells are potent antigen-specific suppressors of immune reactions able to keep CTLs inactive (Togashi et al. 2019). Consequently, one way of escaping immune attacks is to prevent the maturation of DCs.

Cancer induced tolerance is antigen specific, meaning that it does not suppress the immune system systematically (Waldman et al. 2020). Exosomal MHC complexes seem to contribute to the antigen-specific immunosuppression (Düchler et al. 2019). MHC molecules are present not only on the cell surface, but also on exosomes and the level of MHC molecules on the exosomal surface corresponds to the expression of the same molecules on the parent cells (Joo et al. 2020; Wieckowski et al. 2009).

In this study we follow the strategy used by cancer cells to create immunosuppressive exosomes able to shut down unwanted immune reactions presumably in antigen-specific manner. For that purpose, we transfected exosomes with immunosuppressive miRNAs to generate tolerogenic vesicles. For loading exosomes, we choose miR-494 and miR-146a which are described to bear immune suppressive potential (Liu et al. 2012; Mastroianni et al. 2019; Tang et al. 2020). MiR-494 was reported to enhance immature myeloid cells (MDSC) recruitment in cancer environment resulting in immune response suppression (Liu et al. 2012). In addition, miR-146a is defined with immune suppressive ability by inhibiting differentiation of immune cells and anti-inflammatory functions (Lu et al. 2021; Mastroianni et al. 2019; Self-Fordham et al. 2017; Taganov et al. 2006). Small EVs (sEVs) including exosomes were isolated from culture supernatants of monocytic THP-1 cells or healthy donor peripheral blood mononuclear cells (PBMCs), characterized, and transfected by in vitro electroporation with miRNA mimics. After exposure of immature DCs (iDCs) to the electroporated sEVs, changes in the expression of MHC molecules and co-receptors necessary for the efficient activation of immune responses were measured by flow cytometry.

## Materials and Methods

### Cell Culture

The human monocytic leukaemia cell line THP-1 was cultured in RPMI 1640 medium supplemented with 2 mM L-glutamine, 100 IU/ml penicillin, 100 µg/ml streptomycin, 10% fetal bovine serum (FBS), 0.05 mM β-mercaptoethanol and 1 mM sodium pyruvate. For exosome production, cells were cultured in sEV-depleted medium: to eliminate serum sEVs, the 10% FBS containing medium was subjected to ultracentrifugation at 100,000×*g* for 2.5 h (10 °C). Serum-derived sEVs were stored and used as negative controls in some experiments. PBMCs were isolated from buffy coats collected from healthy donors in the Regional Centre for Blood Donation and Haemotherapy by density gradient centrifugation. Human blood derived monocytes were isolated using anti-human CD14 MicroBeads (Miltenyi Biotec, Bergisch Gladbach, Germany) following manufacturer’s protocol. Isolated cells were cultured in AIM V serum free medium (Thermo Fisher Scientific Inc., Waltham, MA, USA) and differentiated to the DCs with granulocyte–macrophage colony-stimulating factor (GM-CSF; 100 ng/ml) and IL-4 (100 ng/ml). Before the addition of sEVs, the cells were activated with lipopolysaccharide (LPS; 100 ng/ml) for 4 h. The methodology of THP-1 cells differentiation was established and described in our previous study Czernek et al. (2015), where we performed a phenotypic analysis of differentiated THP-1 cell line. We confirmed the differentiation of THP-1 cells by detection of characteristic surface markers for macrophages, immature and mature DCs using flow cytometry.

### Isolation of sEVs

sEVs were isolated by differential centrifugation. Briefly, culture medium was centrifuged at 400×*g* for 4 min to remove cell debris. The supernatant was subsequently centrifuged at 10,000×*g*, followed by ultracentrifugation at 100,000×*g* for 2.5 h at 4 °C using pollyalomer tubes (SW 41 Ti rotor, Beckman Coulter Ultracentrifuge L8-80 M). Then, the supernatant was discarded and the sEV pellet obtained was washed with phosphate buffered saline (PBS) by ultracentrifugation under the same conditions. The obtained sEVs were resuspended in PBS and the protein concentration was determined by the Bradford assay (BioRad Laboratories, Hercules, CA, USA) with bovine serum albumin serving as standard.

### Western Blot

Whole cell lysate was prepared from THP-1 cells or THP-1-derived sEVs using RIPA buffer (50 mM Tris–HCl pH 7.4, 150 mM NaCl, 0.1% Triton X-100, 2 mM EDTA, 0.1% SDS) in the presence of protease-inhibitor cocktail (Sigma-Aldrich, St. Louis, MO, USA). The protein concentration of the lysates was determined by Bradford assays. Twenty or 40 ug of protein for cell lysates or exosomes, respectively, were used for Western Blot analysis.

Protein samples were separated on 12% polyacrylamide gels and transferred onto immobilon transfer membranes (Cat# IPVH00010, Merck Millipore, Burlington, MA, USA). The membranes were blocked with 5% bovine serum albumin (BSA) in TBS (20 mM Tris, pH = 7.6; 150 mM NaCl) for 1 h at room temperature (RT), and then incubated with primary antibodies in TBST (TBS with 1% BSA and 0.1% Tween 20) overnight at 4 °C. The next day, membranes were washed three times with TBST and incubated in species-specific, horseradish peroxidase (HRP)-conjugated secondary antibodies for 1 h at RT. After three washing steps with TBST the chemiluminescence substrate was added (Clarity Western ECL Substrate, BioRad Laboratories, Hercules, CA, USA). Chemiluminescence was visualized on the Alliance Q9 Advanced (UVITEC). The following primary antibodies were used: anti-Calnexin (Cat# C4731, 1:1000, Sigma-Aldrich, St. Louis, MO, USA), anti-TSG101 (Cat# sc-7964, 1:1000, Santa Cruz Biotechnology, Dallas, TX, USA), and anti-Alix (Cat# sc-271975, 1:1000, Santa Cruz Biotechnology, Dallas, TX, USA). The following HRP-conjugated secondary antibodies were used: Rabbit anti-Mouse (Cat# P02060, 1:5000; Agilent Dako, Santa Clara, CA, USA), and Goat anti-Rabbit (Cat# P0448, 1:5000; Agilent Dako, Santa Clara, CA, USA).

### Flow Cytometry

#### Extracellular Vesicle Analysis

sEVs were characterized by bead-assisted flow cytometry using antibodies against the classical exosome-marker CD63. Flow cytometric analysis was performed by adsorbing sEVs onto aldehyde-sulphate latex beads (3.8 µm size, Cat# A37304, Life Technologies, Carlsbad, CA, USA) for 20 min at RT. The remaining binding sites on the beads were blocked with high concentration of BSA (1 mg/ml), followed by glycine (100 mM), washed twice with flow cytometry (FACS) buffer (PBS with 1% BSA and 0.1% NaN3) and incubated with PE-conjugated monoclonal antibody for CD63 (Cat# 556,020, BD Biosciences, USA). Incubation was performed for 1 h in dark at RT, the beads were washed again with cold FACS buffer, and analysed with BD CellQuest Pro on a FACS Flow Calibur (Becton Dickinson, Poland).

#### Cell Staining

For the analysis of cell surface receptors, the cells were harvested in ice-cold PBS. Adherent cells were detached from the plastic by gentle pipetting. All immune-labelling steps were performed on ice for 1 h in the dark. The specific antibodies (BD Pharmingen, USA) were used to analyse the expression of CD86 and MHC receptors via a FACS Calibur flow cytometer (Becton Dickinson, Poland) using CellQuest Pro software (BD). HLA-I was detected with PE-labelled mouse–anti-human HLA–ABC (Cat# 555,553; BD Pharmingen USA), HLA-II with FITC-labelled mouse anti-human HLA-DR (Cat#555,560, BD Pharmingen, USA) and CD86 with APC-labelled mouse anti-human CD86 (Cat#555,660, BD Pharmingen, USA). Corresponding fluorescence label-conjugated isotype controls were used in this experiment.

#### Cytometric Bead Assay

The PBMC-conditioned media were tested for the presence of soluble cytokines. Samples were measured using Cytometric Bead Arrays (BD Biosciences, USA) for IL-10 (Cat# 558,274, BD) and TGF-β (Cat# 560,429, BD) according to the manufacturer protocols. All samples were analysed using a BD FACS Calibur flow cytometer (BD Bioscience, USA) and the FCAP Array software (BD version 3.0). A ten-point standard curve was included.

### Transmission Electron Microscopy

For the transmission electron microscopy (TEM) analysis, sEVs or post-electroporated sEVs were placed on formvar-carbon coated electron microscopy grids (FCF200-Cu-50, 200 mesh, Electron Microscopy Sciences, Hatfield, PA, USA) and left to adhere for 20 min. Next, the vesicles were fixed in 1% (*v*/*v*) glutaraldehyde and contrasted in 2% (*w*/*v*) aqueous uranyl acetate (Polysciences) in water for 10 min. The sEVs were examined using a transmission electron microscope (Talos F200X, 200 kV).

### Electroporation

Twenty µg sEVs from THP-1 or healthy donor PBMCs were mixed with miRNA mimics at a final concentration of 0.4 µM in electroporation buffer (Phosphate-free buffer: 125 mM NaCl; 5 mM KCl; 1,5 mM CaCl2; 10 mM Glucose; 20 mM HEPES pH 7.4; 50 mM Trehalose; 1 mM EDTA), in a final volume of 200 uL, then incubated for 10 min on ice, transferred to pre-cooled cuvettes and electroporated (one pulse with 350 V and 150 µF using Gene Pulser Xcell (BioRad Laboratories, Hercules, CA, USA), after which the samples were incubated on ice for a further 20 min. To remove unincorporated miRNA, the samples were diluted with 11 ml PBS and submitted to ultracentrifugation at 100 000×*g* for 2.5 h. The pellet of exosomes was then resuspended in 200 µL of sEV-depleted medium and added to the cells (200 µL per well in a 12-well plate, seeding concentration of 50.000 per well). Cells were incubated in sEV-depleted medium at 37 °C for 72 h after the addition of miRNA-loaded sEVs or naked control miRNAs.

### Quantitative Real-Time Reverse Transcriptase PCR

TaqMan^®^ Small RNA Assays (Applied Biosystems by Life Technology Corporation, Van Allen Way, Carlsbad, CA, USA) was used to quantify miR494-3p, -5p and U6 in THP-1 cells after incubation cells with synthetic miR494 duplexes with or without exosomes. Total RNA concentration was determined by UV absorption using ND-1000 spectrophotometer (NanoDrop). In the first step, the RT reaction (master mix) was prepared using the TaqMan^®^ MicroRNA Reverse Transcription Kit according assay protocol. Real-time PCR reactions were prepared on 96-well plate (Roche Diagnostics GmbH, Mannheim, Germany). Component volume per 20 μL real-time PCR reaction: 1 μL of TaqMan^®^ Small RNA Assay (20 ×)–TaqMan^®^ probe and primers for miR494-3p, -5p and U6; 1.33 μL of product from RT reaction; 10 μL TaqMan^®^ Universal PCR Master Mix II (2 ×), and 7.67 μL nuclease-free water using the following parameters on LightCycler^®^96 instrument (Roche Diagnostics GmbH, Mannheim, Germany): 95 °C per 10 min (enzyme activation); 95 °C per 15 s (denature) and 60 °C per 60 s (anneal/extend)—40 cycles. After the reaction LightCycler^®^96 software 1.1 was used to analysed amplification results and GraphPad Prism 4.0 (GraphPad Software, San Diego, CA, USA) was used for graph preparation.

### Chemical Synthesis of miRNAs Mimics Oligonucleotides

The unmodified RNA oligomers were synthesized in Division of Bioorganic Chemistry of CMMS PAS Łódź by dr Anna Maciaszek using Gene World automated DNA/RNA synthesizer at a 0.2 μmol scale, with commercially available LCA–CPG supports (Biosearch Technologies, Inc., Petaluma, CA) and the standard phosphoramidite monomers (Glen Research, Sterling, VA, USA). The phosphoramidites were dissolved in anhydrous CH3CN at concentration of 0.07 M, and a 0.25 M solution of 5-Benzylmercapto-1H-tetrazole in anhydrous CH3CN was used as an activator. Oligonucleotides were deprotected and isolated using a binary Varian HPLC system, consisting of two PrepStar 218 pumps and a ProStar 325 UV/VIS detector set at 260 nm. A reverse phase HPLC column (PRP-1, C18, 7 μm, 305 × 7 mm, Hamilton, Reno, NV, USA) was eluted with a 1% min − 1 gradient of CH3CN in 0.1 M TEAB (pH 7.3) at a flow rate 2.5 ml min − 1. The miR494 and miR146a mimics sequences: miR494-5p 5ʹ-AGGUUGUCCGUGUUGUCUUCUC-3ʹ; miR494-3p 5ʹ-UGAAACAUACACGGGAAACCUC-3ʹ; miR146a-5p 5ʹ-UGAGAACUGAAUUCCAUGGGUU-3ʹ; miR146a-3p 5ʹ-CCUCUGAAAUUCAGUUCUUCAG-3’.

### Statistical Analysis

All the results are expressed as mean end ± SE (standard error) and analysed by the Statistica ver. 8.0 software (StatSoft Inc., Tulsa, OK, USA). The Student’s *t* test was used to calculate the differences between data of two independent groups. Multiple means of groups were determined using one-way ANOVA test with post-hoc analysis—Tukey honestly significant difference. The Levene’s test and Brown–Forsythe test were used to verify homogeneity of variance. A value of *P* < 0.05 was considered statistically significant.

## Results

### Isolation and Characterization of sEVs

**Fig. 1:**
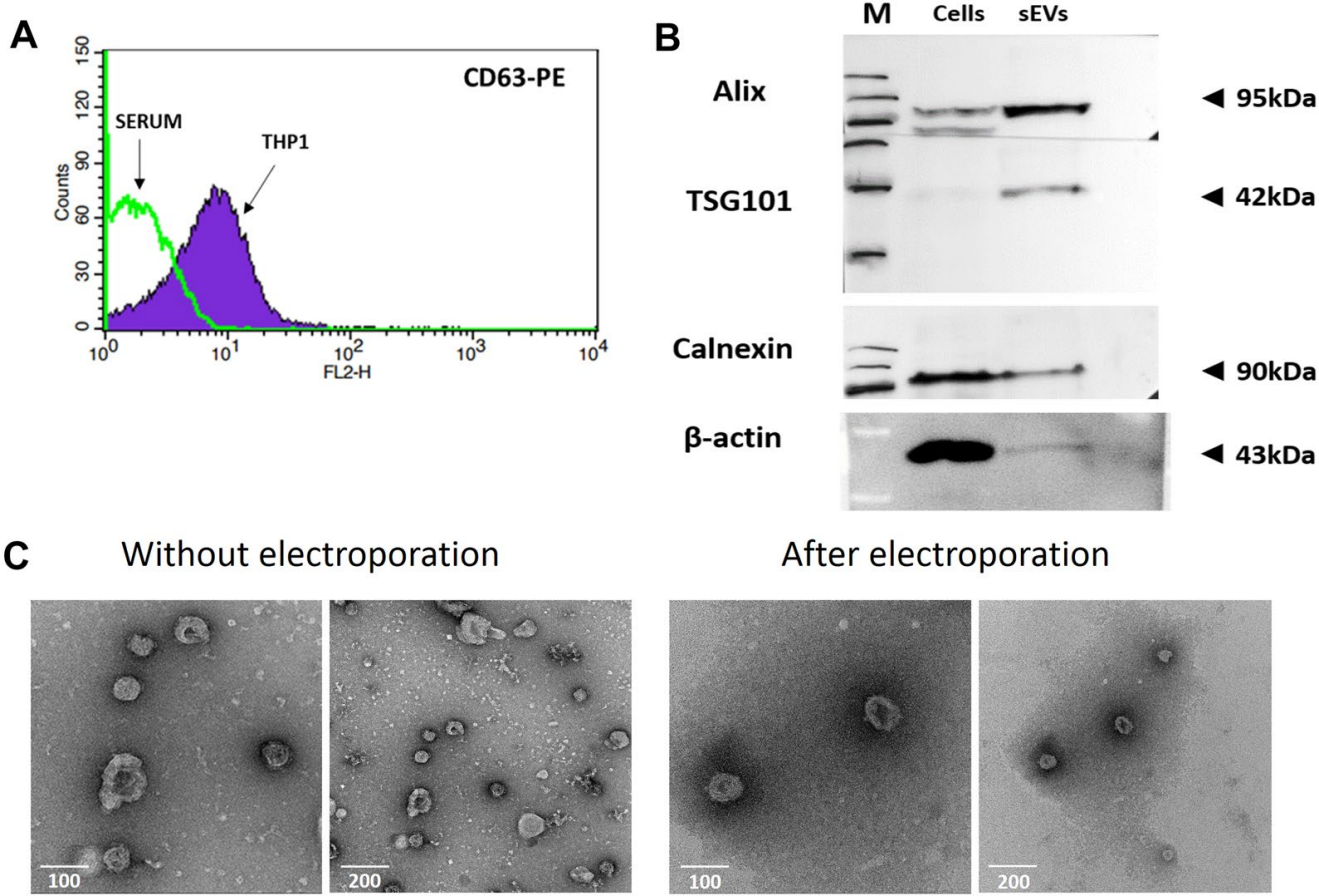
Characterization of the isolated sEVs. **A** Flow cytometry analysis of sEVs. The vesicles were isolated from calf serum (negative control), or cell culture supernatants of the THP-1 cell line, adsorbed to latex beads and stained with PE-antiCD63 antibodies. **B** Western blot analysis of Alix, TSG101 and Calnexin in lysates of THP1 cells and THP-1-derived sEVs. β-Actin was used as loading control. M-PageRuler Plus Prestained Protein Ladder, from the top 250; 130; 95; 72; 55; 36 kDa. **C** Morphological analysis of isolated sEVs using TEM. THP-1-derived sEVs visualized by TEM in the absence of electroporation or post electroporation as indicated. Cup-shaped structures, with 40–100 nm in diameter and contrasting dark halo were detected in both samples. No morphological differences are observed. Scale bars equal 100 or 200 nm

sEVs were isolated and purified from cell culture supernatants of THP-1 cells after the cultures reached a cell density of 0.7–1 × 10^6^ cells/ml. sEV-depleted medium was used for sEV production. In case of PBMCs, cells isolated from buffy coats of healthy volunteers by Ficoll cushion centrifugation were cultivated in high density (1 × 10^7^ cells/ml) overnight in serum-free medium. The next day, sEVs were isolated from the medium by serial centrifugations. For the characterization of the obtained vesicles, flow cytometry, Western blots and TEM were employed (Fig. 1). As shown in Fig. 1A, THP-1-derived sEVs stained positive for the exosome marker CD63.

Western blotting revealed that Alix and TSG101 were enriched in vesicle lysates, while Calnexin was predominantly found in cell lysates (Fig. 1B). The most commonly used technique to incorporate siRNA or miRNA into sEVs is electroporation. This method destabilizes the vesicle membrane and allows siRNA or miRNA to enter the vesicles. The integrity of the membrane is then recovered. However, aggregation of sEVs and RNA during electroporation has previously been reported that may result in suboptimal loading. Aggregation could be prevented using an optimized buffer containing trehalose. This disaccharide was able to aid in maintaining the structural integrity and could inhibit the aggregation of sEVs extracted from adipose-derived stem cells (Johnsen et al. 2016). TEM images taken before and after electroporation revealed that the EVs were approximately spherical in shape and size, suggesting that electroporation did not affect vesicle morphology. As electroporation was carried out in the trehalose containing buffer, no increase in aggregation could be observed (Fig. 1C).

### Detection and Quantification of miRNA after Electroporation of sEVs

**Fig. 2:**
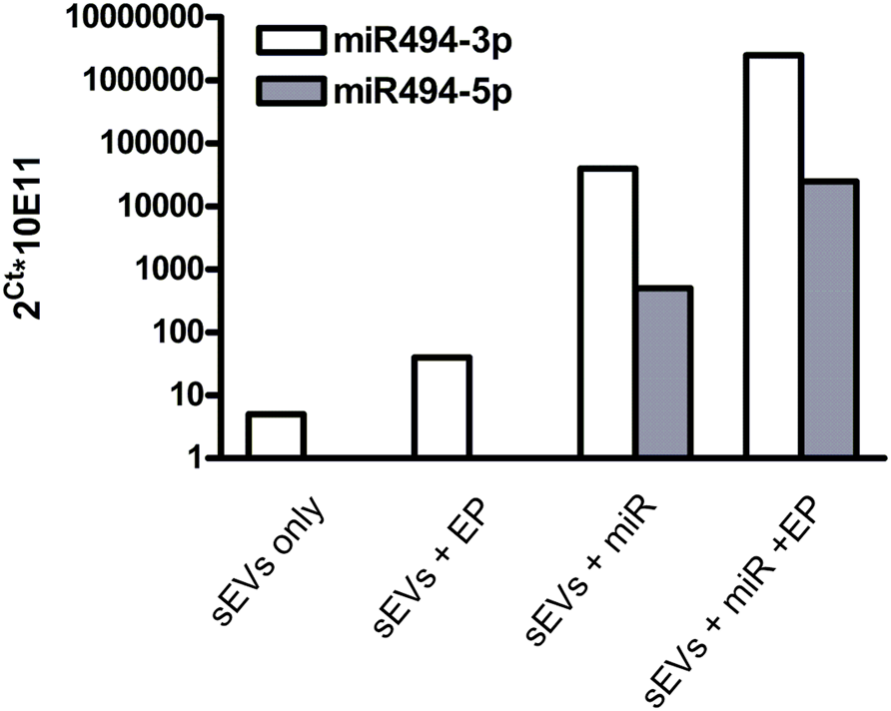
Quantification of miR494 after electroporation. RNA was isolated from sEVs after electroporation with miR494 mimics and used for reverse transcription and real-time PCR to quantify both strands of the miR494 RNA duplex. The number of miRNA molecules was estimated by relating the obtained values to a standard curve created with synthetic miR494. This experiment was done only once

Electroporation leads to superior loading of siRNA in comparison with chemical transfection (Wahlgren et al. 2012). To demonstrate successful loading of miR494 into sEVs, total RNA was isolated after EP of the miR494 mimic and miR494 was quantified by qRT-PCR (Fig. 2).

Mir494-3p is the strand that is naturally occurring inside cells and sEVs, and was, therefore, also detected in sEVs without electroporation, while the miR494-5p strand became visible only in sEV samples exposed to the miR494 mimics (Fig. 2). Surprisingly, addition of a mixture of miR494 mimics and sEVs (without electroporation) also resulted in a substantial number of isolated miR494 molecules, although the mixture was subjected to ultracentrifugation to remove unincorporated miRNAs. A possible explanation is binding of the mimics to the vesicles’ surface and this association seemed to outlast the purification step by ultracentrifugation. Following electroporation, the level of miR494-3p was about ten times higher than without this loading procedure.

### miRNA Mimics are Efficiently Delivered to Target Cells

In untreated THP-1 cells, miR494-3p and miR494-5p were almost undetectable. After incubation of the cells with miR494 mimic loaded sEVs, a clear enrichment of miR494-3p became measurable, while miR494-5p did not accumulate. The highest amount of miR494-3p was detected after direct electroporation of the cells with the mimic; however, also in this case, the level of the miR494-5p strand was much lower (about 1%, data not shown). Apparently, the 5p-strand was rapidly degraded inside the cells. The control including sEVs and mimics without electroporation also gave a reproducible signal for miR494-3p indicating again that the double stranded RNA can enter the cells with the help of the vesicles even without being internalized.

### miR494 Transported by sEVs Slightly Reduced the Expression of MHC-I and CD86 Molecules on THP-1 DCs

In our previous study (Czernek et al. 2015), we performed a phenotypic analysis of the THP-1 cell line cell after induced differentiation. The mature DCs could be distinguished from iDCs, macrophages and monocytes by high expression of MHC I (HLA-A,B,C). In addition, the expression of CD86 in differentiated cells was 6–17 times higher than in monocytes.

**Fig. 3:**
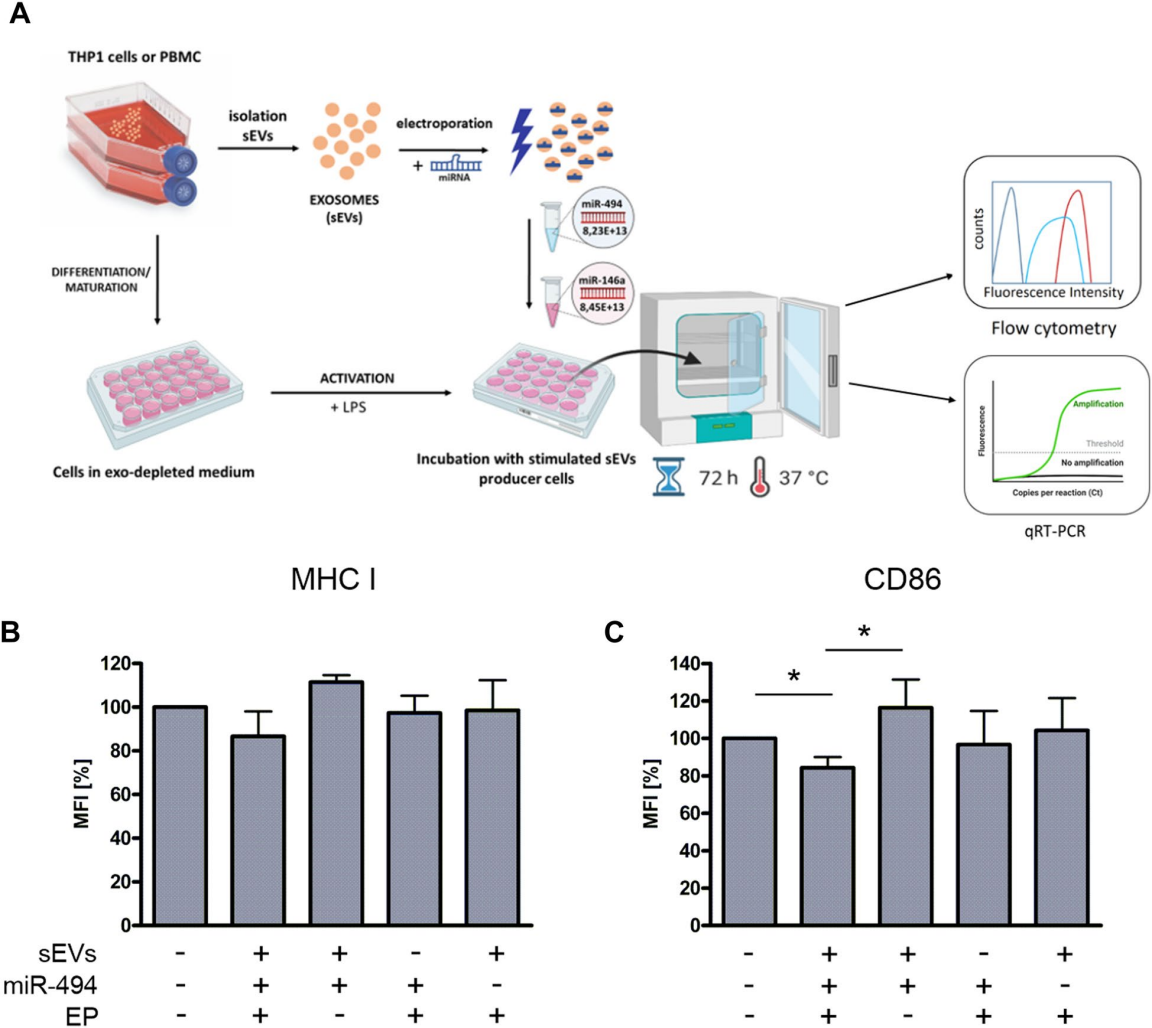
MHC class I and CD86 expression levels in THP-1 cells. **A** General schema of the experiments: sEVs were isolated from THP-1 monocytic cells, loaded with miR494 and added back to THP-1 cells which were differentiated into immature DCs and incubated with LPS to induce further maturation. During the maturation process, sEVs loaded with miR494 or controls were added as indicated. Also a control with the addition of miR494 without sEVs is shown. **B** Mean fluorescence intensities (MFI) of MHC-I stainings. **C** MFI of CD86 stainings. Graph represents results from four independent experiments. EP—electroporation, sEVs—small extracellular vesicles. *P ≤ 0.05

We investigated whether miR494 mimics loaded sEVs could interfere with the maturation of DCs. MiRNAs are involved not only in cancer progression and development, but also in the control of immunological processes, such as differentiation and activation of immune cells (Otmani and Lewalle 2021; Peng and Croce 2016). THP-1 monocytic cells were differentiated toward iDCs by incubation with GM-CSF and IL-4. After 3 days, maturation was induced by the addition of LPS in the presence or absence of sEVs (Fig. 3A). Negative controls included: sEVs plus miR494 mimics without electroporation, miR494 electroporated directly into the cells, and miR494 or sEVs as single agents, both after electroporation. Despite no statistical significance we can still observed some tendency, which in our opinion is relevant. The combination of sEVs with miR494 without electroporation increased the mean fluorescence levels of MHC I and CD86 surface proteins, while application of electroporation created sEVs which were able to reduce the mean fluorescence intensities (MFI) of these receptors by about 15% or 25% compared to the untreated control cells or the non-electroporated sEVs, respectively (Fig. 3B, C). More research is needed to clarify this phenomenon and highlight the differences, which may be masked by the multiple cellular processes taking place in the cell.

### miR494 Transported by sEVs Reduced the Expression of MHC I, MHC II and CD86 Molecules on PBMC-Derived DCs

**Fig. 4:**
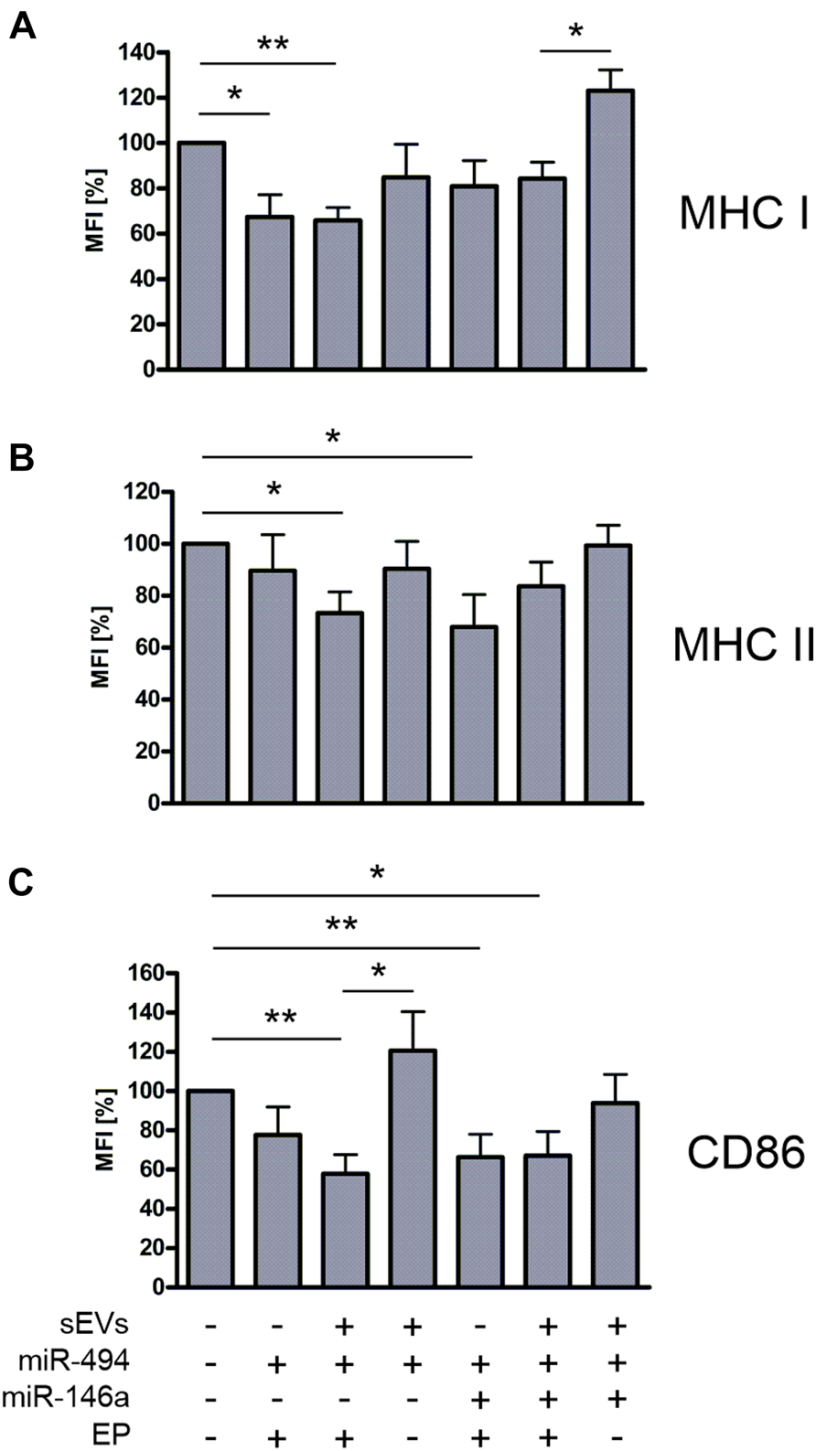
MHC and CD86 expression levels in PBMC derived DCs. sEVs were isolated from PBMCs, loaded with miR494 and miR146a mimics. CD14^+^ cells were differentiated into immature DCs and incubated with LPS to induce further maturation. During the maturation process, sEVs were added as indicated. **A** Mean fluorescence intensities (MFI) of MHC-I stainings. **B** MFI of MHC-II stainings. **C** MFI of CD86 stainings. Graph represents results from five independent experiments. EP—electroporation, sEVs—small extracellular vesicles. *P ≤ 0.05; **P ≤ 0.01

Next, the influence of miRNA mimics loaded sEVs on the maturation of DC cells was tested using PBMC-derived DCs. Monocyte-derived DCs show high expression of MHC I and MHC II molecules (Lühr et al. 2020; Steinman 1991). CD14^+^ cells were isolated from the PBMCs of healthy donors and differentiated toward iDCs by incubation with IL-4 and GM-CSF (Castiello et al. 2011). After 3 days, maturation was induced by the addition of LPS in the presence or absence of sEVs. Besides miR494 mimics, in some experiments also miR146a mimics were included. The combination of sEVs with miR494 without electroporation increased the mean fluorescence levels of CD86. Application of electroporation created sEVs which were able to reduce the MFI of MHC I, MHC II and CD86 by about 35%, 25%, and more than 40% compared to the untreated control cells, respectively (Fig. 4). The addition of miR-146a did not further reduce the MFI of the measured surface receptors. The direct electroporation of miR-494 into the DCs (Fig. 4, second columns from left side) also resulted in reduced surface expression of the three receptors which was significant in case of MHC I and CD86.

### Measurement of the Production of Immunosuppressive Cytokines

Tolerogenic APCs were shown to produce immunosuppressive cytokines, such as TGF-β and IL-10 (Tang et al. 2020). Using a cytokine cytometric bead array and flow-cytometry panel, we investigate the expression of IL-10 and TGF-β cytokines expressed by PBMC cells after exposure to miRNA-loaded exosomes. The FCAP Array software was used to convert the fluorescent MFI values into cytokine concentrations by correlation them with appropriate standard curves. No IL-10 or TGF-β could be detected in the cell culture supernatants (results not shown).

## Discussion

Exosomes are one of the tools used by cancer cells to develop immunosuppression (Czernek and Düchler 2017; Kugeratski and Kalluri 2021). Cancer-induced tolerance often does not suppress the immune system systematically, but shuts down the specific immune reaction directed toward the TAAs (Huang et al. 2013). In our previous work, we presented evidence for antigen-specific immunosuppression mediated by melanoma derived sEVs (Düchler et al. 2019). Cancer derived sEVs can transport antigenic peptides in complexes with MHC molecules to APCs, a process called MHC cross-dressing (Ravindranath et al. 2021; Zeng and Morelli 2018). The peptide–MHC complexes confer the antigen specificity to the APCs for their interaction with T cells, while other sEV components decide about stimulation or suppression of immune responses.

Besides cancer cell derived exosomes, also vesicles released by normal healthy cells can have stimulatory or tolerogenic properties. For instance, exosomes derived from tolerogenic DCs suppress immune reactions via the induction of Treg cells, while mature DC-derived exosomes are able to stimulate immunity (Engeroff and Vogel 2022). Lee et al. (2021) used tolerogenic DC-derived exosomes to shut down inflammation in a rheumatoid arthritis model. The exosomes were taken up by mature DCs in the inflamed joints and down-regulated CD40 and inflammatory cytokines. Recently, allograft derived sEVs were recognized as crucial components to mediate acute rejection (Benichou et al. 2020; Gonzalez-Nolasco et al. 2018; Zeng and Morelli 2018). In a process called semi-direct pathway of allorecognition, sEVs from donor cells are taken up by recipient APCs to present donor MHC antigens to alloreactive *T* cells. On the other hand, donor derived exosomes carrying MHC molecules were recognized for their ability to prolong allograft survival in rodents (Morelli 2006; Pêche et al. 2006).

There are very few reports on sEV engineering with the aim to increase their tolerogenic potential, although sEVs are promising vehicles for miRNA delivery with better safety profiles than polymer-based particles, cationic lipids, viral vectors or non-viral vectors which are divided into six categories: inorganic material-based delivery systems, lipid-based nanocarriers, polymeric vectors/dendrimer-based vectors, cell-derived membrane vesicles and 3D scaffold-based delivery systems (Chen et al.^2021^; Fu et al. 2020; Kugeratski and Kalluri 2021; Taghikhani et al. 2020). Of course, all these methods have both advantages and disadvantages (viral-based delivery vectors have high to low carcinogenic potential, are immunogenic but have stable transgenic expression vs non-viral-based delivery vectors which are non-immunogenic but could be cytotoxic). In all of these delivery systems only EVs have high packaging capacity, are non-immunogenic and deliver cargo tissue-specific (Dasgupta and Chatterjee 2021). For example, Ohno et al. (2013) reported the targeted delivery of sEVs transfected with let-7a miRNA to epidermal growth factor-overexpressing breast cancer cells in a mouse xenograft model. In alternative approaches, the EV producer cells were manipulated to create vesicles with immunosuppressive properties (Elashiry et al. 2021; Schülke 2018).

However, naturally occurring, unmodified exosomes have been used to suppress unwanted immune reactions in inflammatory diseases, autoimmunity and transplantation biology. These vesicles were derived from mesenchymal stem cells, and suppressed inflammatory responses (Harrell et al. 2013; Riazifar et al. 2019), promoted the development of myeloid cells into immunosuppressive ones (Biswas et al. 2019; Gurunathan et al. 2019b; Shahir et al. 2020), and contributed to cross-priming of plasmacytoid DCs (Fu et al. 2020). The cord blood is another source of tolerogenic sEVs which were shown to induce an anti-inflammatory phenotype in macrophages (Rodrigues et al. 2021). The tolerogenic activity of this kind of EVs is not antigen-specific. In contrast, sEVs derived from Treg cells can confer antigen-specific immunosuppression, a process that may include miRNA transfer (Tung et al. 2018) or the surface transport of IL-35 (Sullivan et al. 2020). Tumour-derived exosomes (TDEs) were also shown by Hosseini et al. (2021) to carry several bioactive molecules (HLA-G, COX-2, PGE2, TGF-β, IL-6, HSP70, HSP72) that can interfere with the maturation of DCs, thus decreasing their capability in inducing effective anti-tumour responses. Moreover, others have shown that TDEs can alter the function of well-differentiated mature DCs. According to the published data, the interaction/uptake of tumour-derived exosomes by mature DCs renders them to an immunosuppressive phenotype, which thereby can improve tumour immune evasion (Hoseini et al. 2021).

We aimed at engineering sEVs for potential therapeutic purposes making them tolerogenic through the transfection with miRNAs. A big advantage in the potential practical application of this strategy is based on the fact that identification of the TAAs and the antigenic peptides derived thereof is not required. The sEVs are isolated from the particular target tissue of immune attack, made tolerogenic, and are fed to APCs which are thus routed toward a suppressive phenotype. In this study, the sEVs were equipped with immunosuppressive miRNA mimics to confer the tolerogenic character. First, we demonstrated successful isolation of sEVs, their efficient loading with miR494 mimics, and transport of miR494 into THP-1 cells. miR-494 was chosen, because it was described as essential for the accumulation and activity of MDSCs (Liu et al. 2012). In some experiments, miR146a was added as a second miRNA. MiR146a is a major regulator of innate immune responses (Saba et al. 2014) and contributes to myeloid tumorigenesis (Testa et al. 2017). In the melanoma microenvironment, miR146a was identified as a negative regulator of immune activation (Mastroianni et al. 2019). MiR146a is widely expressed in Tregs and promotes their ability to restrict *T* helper cell type 1 responses (Khorrami et al. 2017; Lu et al. 2021). Furthermore, miR146a was shown to down-regulate CD80 and CD86 in DCs and to increase the secretion of the immunosuppressive cytokines TGF-β and IL-10 (Tang et al. 2020). In our experiments, no IL-10 or TGF-β could be detected after miR-146a transfer. Chen et al. (2011) confirmed that miR-146 partly induces the maturation of monocyte-derived DCs. The mRNA and protein levels of CD40, CD80, and CD86 of DCs were upregulated in response to incubation with miR-146a inhibitor, meanwhile, down-regulated in response to miR-146a mimics which is in line with our observations (Chen et al. 2011).

As a readout for the tolerogenic activity of our engineered sEVs we measured the expression levels of MHC I, MHC II and CD86 receptors in the recipient DCs (Dudek et al. 2013). While unmodified sEVs used as a control did not diminish the expression of these molecules but rather showed a slightly enhancing effect, the miRNA mimics loaded sEVs were able to reduce the expression levels of these surface proteins. The reduction was visible in the THP-1 cell line, and even more pronounced in DCs derived from the blood of healthy donors. The direct comparison of modified versus unmodified sEVs allows us to conclude that indeed the minimal engineering we performed substantially changed the character of the vesicles making them more immunosuppressive.

## Conclusion

The present study provides evidence that sEVs can be used to create tools for immunosuppression similar to the strategies used by tumours. This kind of approach may result in an antigen specific immunosuppression when the sEVs are isolated from the cells/tissues that should be spared from immune attacks. Our findings may contribute to the development of powerful tools suitable for antigen-specific down-modulation of harmful immune responses.
